# Age-Related Differences and Reliability of a Field-Based Fitness Test Battery in Young Trained Footballers: The Role of Biological Age

**DOI:** 10.3390/life14111448

**Published:** 2024-11-08

**Authors:** Jose Jimenez-Iglesias, Oliver Gonzalo-Skok, Mario Landi-Fernández, Alejandro Perez-Bey, Jose Castro-Piñero

**Affiliations:** 1 Galeno Research Group, Department of Physical Education, Faculty of Education Sciences, University of Cadiz, 11519 Puerto Real, Spain; joselete.jimenez@gmail.com (J.J.-I.); mariolandif04@gmail.com (M.L.-F.); jose.castro@uca.es (J.C.-P.); 2 Sport Science Department Cádiz C.F., Cádiz C.F., 11519 Cádiz, Spain; 3 Department of Communication and Education, Universidad Loyola Andalucía, 41014 Sevilla, Spain; 4 Instituto de Investigación e Innovación Biomédica de Cádiz (INiBICA), 11009 Cádiz, Spain; alejandro.perezperez@uca.es

**Keywords:** soccer, cardiorespiratory fitness, muscular strength, change-of-direction ability, peak height velocity, reproducibility

## Abstract

Background: the purpose of this study was to analyze the reliability of a field-based fitness test battery in young trained football players, according to biological age. Methods: 197 young trained football players (12–19 years old) participated in the study. We measured anthropometric measurements (i.e., height, sitting height, length leg, and body mass), a bilateral vertical jumping test (CMJ), a progressive loading test of squats and hip thrust, acceleration and speed tests (10 m and 30 m sprint tests), a change-of-direction ability test (V–cut test), and a cardiorespiratory fitness test (30–15 intermittent fitness test). Statistical data are shown as the mean ± standard deviation by PHV group in tests and retests. Test reliability was assessed through the intraclass correlation coefficient (ICC), with an ICC above 0.9 being considered high. To evaluate accuracy and repeatability, standard error of measurement, coefficient of variation, and minimum detectable change at 90% were determined and Bland–Altman diagrams were used, establishing a statistical significance of *p* < 0.05. Results: All of the tests showed non-significant differences between the test and retest in the pooled sample (*p* > 0.05). Furthermore, all of them presented a trivial effect size (<0.2) and high intraclass correlation coefficients (>0.9), which indicates the high reproducibility of the tests, despite some of them presenting a significant difference between trials (i.e., the CMJ, 10 m sprint, V–cut, and squat tests). Low measurement errors were found for all tests (coefficient of variation [CV] = 4.39–9.39), except for the CMJ and the progressive loading test for squat and hip thrust exercises (CV = 12.2–21.11). Similar results were found irrespective of biological age group. Conclusions: All tests were reliable for the pooled sample as well as for the biological age groups.

## 1. Introduction

Football performance is influenced by multiple components such as the technical, tactical, and psychological, as well as fitness [1]. Fitness is especially important to achieve competitive success according to the trend in today´s football [2]. There are numerous fitness components which have a direct relationship with increased performance in both adult and young football players [3], such as anthropometric variables [4], lower-body muscular strength [5], sprinting and change-of-direction (COD) ability [5,6], and/or cardiorespiratory fitness [7]. Thus, it seems interesting to assess the level of these components to know the fitness status of players at different ages for decision making related to the training process.

There are two variables to consider with respect to a player’s physical performance as it is related to age: chronological and biological age [8]. Chronological age refers to the period from the date of birth to the current day, which makes the individual belong to a certain competitive age group (e.g., U–13, U–15) [9]. Despite what had been widely believed, the relative age effect does not seem to have an effect on the performance of soccer players. Even though in professional soccer there is a greater presence of players from the first quarter of the year, the players (strikers) with the highest market value today are mostly from the fourth quarter of the year, so the lack of players born later in the year in youth and professional soccer is not linked to a lower level of talent, but to physiological and socio-cultural factors [10]. On the other hand, biological age refers to the biological status of players, understood as the point of development towards adulthood [11]. It varies according to each biological system and in terms of status, timing, and tempo [11,12]. Biological age plays an important role in somatic development and the evolution of the variables that determine performance in young football players [12]. It has been shown that more mature football players appear to have higher performance in terms of anthropometric variables, jumping ability, lower-body and maximal muscular strength, cardiorespiratory fitness, and even technical skills [8]. In fact, a higher biological age seems to give a player a greater chance of being signed by a professional football academy in comparison to another player of the same chronological age but who is less mature [9]. It results in the premature discarding of players who could develop high performance levels when they reach their full biological development [13]. For these reasons, it is important to know the inter-individual differences in performance that may arise as a result of the maturity level of young football players [14]. It is also important to know an individual’s performance relative to his/her peak height velocity (PHV), which is directly related to the development of a player’s fitness [15]. Age at PHV is strongly associated with maturational age, providing an accurate benchmark of growth during puberty from the rate of growth of body dimensions [16]. If we really want to make good decisions with respect to the fitness of our players, it is crucial to place importance on biological maturation, which has a direct influence on the results of physical tests and, therefore, on the components that determine physical performance in football [17].

When selecting field-based fitness tests, it is important to ensure that they are reliable. A test is considered reliable when it is performed on two or more occasions under the same conditions and very together close in terms of time, and similar results are obtained [18]. Based on scientific evidence, the most widely used field-based fitness tests that assess the above-mentioned components of fitness have proven their reliability in general populations of athletes but not with male soccer players. The muscular strength of the lower-body, evaluated through the progressive loads test for the squat (SQ) and hip thrust (HT), has been widely used to assess these fitness components [19,20]. SQ tests have shown their reliability in general populations of athletes [21], but the HT tests have not. COD ability, evaluated through the V–cut test, and jumping ability, evaluated through the counter movement jump (CMJ) test, are reliable variables for basketball players [22] and for a general population of athletes [23], respectively. Only acceleration and speed through the 10 m sprint test [24,25] and 30 m sprint test [25], cardiorespiratory fitness through the 30–15 intermittent fitness test (30–15) [26], and anthropometric variables [24], have been proven their reliability with populations of young football players. However, despite the influence that biological age could have on physical performance [12], none of the selected tests took this variable into account to check their reliability, with the only exception being the anthropometric variables, jumping ability and acceleration [24], and jumping ability [23], with small samples of 80 and 30 players, respectively. In this study, we hypothesize that it is possible to develop a reliable test battery to assess the physical fitness of young trained soccer players without biological age influencing this reliability.

Therefore, the aim of the present study was to analyze the reliability of a field-based fitness test battery in young trained football players according to biological age.

## 2. Materials and Methods

### 2.1. Participants

A total of 197 young male trained soccer players from U–13 to U–19 (14.74 ± 2.2 years) belonging to the Cadiz C.F. Academy (LaLiga Santander, the 1st Spanish division) volunteered to participate in this study (Supplementary Material Table S1). Participants trained an average of 4 days (75 min per training session) per week plus one official match (90 min), which amounts to an average of 390 min of football per week. All of the players had at least four years of previous experience in football and carried out training sessions at their club for the development of their soccer abilities. At the time of the study, all of the players were competing at regional level in their respective categories. Data collection took place during week 21 of the competitive season after two months of preseason.

The players and their legal guardians were informed in detail of the nature of the study, as well as its objectives, and any doubts that might have arisen were resolved. The present study was conducted in accordance with the Helsinki declaration for research involving human subjects (Declaration of Helsinki II) and was approved by the University of Zaragoza ethics committee, and written consent was obtained from the parents or legal guardians of those under 18 years old.

### 2.2. Experimental Design

We analyzed the reliability of a field-based fitness test battery in young male football players according to biological age. We collected data from anthropometric measurements (height, sitting height, length leg, and body mass) and administered a lower-body strength test (i.e., CMJ test, a test of SQs and HTs through progressive), acceleration and speed tests (i.e., 10 m and 30 m sprint tests), a COD ability test (i.e, the V–cut test), and a cardiorespiratory fitness test (i.e., the 30–15 IFT). Lower-body strength in SQs and HTs was only calculated from U–17 to U–19 because it is a usual part of the training routine at those ages. The 30–15 IFT was also not performed in the U–13 group, as literature says. We did not apply this test because this is not a sensitive stage for the development of this aspect of fitness and it is not a determinant of performance at this age [12]. Likewise, reviews that have been carried out on 30–15 IFT have not found studies where it was applied at these ages [26,27].

**Figure 1:**
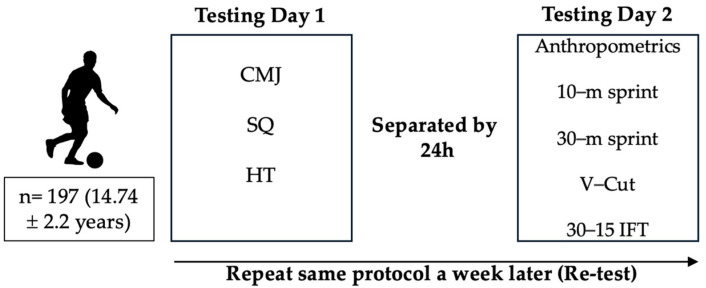
Experimental design diagram. CMJ, counter movement jump; HT, hip thrust; 30–15, 30–15 intermittent fitness test; SQ, squat, V–cut test.

Due to the possible interference of fatigue, the field-based fitness tests were carried out on two different days, separated by at least 24 h. On the first day, we assessed the CMJ test and progressive loading test in the SQ and HT exercises, in that order. On the second day, the following tests were assessed as follows: anthropometric measurements, the 10 m and 30 m tests, the V–cut test, and the 30–15 IFT (Figure 1). They were scheduled so that the players had a recovery session on match day (MD+1) of the microcycle (i.e., low-load football tasks, mobility, manual and compressive therapy, and cryotherapy), followed by 24 h of passive recovery in MD+2. So, the evaluations took place on MD-4 after 72 h of recovery. All players had a similar workload that week. After this first evaluation, the same process was repeated one week later (retest reliability) at the same time of the day (4 p.m. to 8 p.m.) and under the same experimental conditions (~15 °C and ~41% humidity). Players were instructed to maintain their usual nutrition and hydration habits. Two weeks prior to the assessment, players carried out a familiarization session based on the execution of the tests at low–medium intensity, as has been performed in other similar studies [23] (with the exception of the 30–15 IFT), during their warm-up routine twice a week.

### 2.3. Procedures

Prior to the tests, a warm-up of 15 min consisting of jogging (5 min), lower limb dynamic stretches (2–3 min), and lower limb horizontal and vertical explosive actions (4 × 20 m progressive accelerations, and 6 progressive CMJs) was performed with 3 min of passive recovery before starting the tests. All players recovered for 3 min between sets and for 5 min between trials, and the tests were supervised by technical staff and verbal encouragement was provided to players in all of the trials.

#### 2.3.1. Anthropometric Measurements and PHV

We measured height barefoot with the players standing upright and the head in the Frankfurt plane [28] using a stadiometer (SECA 217, Hamburg, Germany) with an accuracy of 0.01 cm. Body mass (BM) was measured barefoot with the player in an anatomical position [28] using a bioimpedance scale (H-BIA, Omrom, model 306C, Hoffman Estates, IL) with an accuracy of 0.1 kg. In addition, the sitting height was obtained, with the players seated on a box with the back straight and the head in the Frankfurt plane [28,29] using a stadiometer (SECA 217, Hamburg, Germany) with a sensitivity of 0.01 cm [29]. Each measurement was taken twice, and the mean was recorded for analysis [3].

The leg length was calculated as the difference between the height and the sitting height, and PHV was calculated using the formula for men provided by Mirwald et al. (2002) [29]. Players were classified according to their PHV as pre < −1 (PHV0 group = −1.86 ± 1.06), circa ≤ ±1 (PHV1 group = 0.51 ± 0.26), and post > +1 (PHV2 group = 1.95 ± 0.63) [30].

#### 2.3.2. Lower-Body Muscular Strength

##### Jumping Ability

CMJ: The test was performed with appropriate footwear on a technical floor in a gym. The test was performed by following the instructions of Darrall-Jones et al. (2015) [31] using a jumping platform (Chronojump-BoscoSystem, Barcelona, Spain). A total of 5 maximal CMJs were performed, the best and worst were discarded, and the mean of the selected three jumps was recorded for analysis [32].

Progressive loading test in SQ and HT: The test was performed with appropriate footwear on a technical floor in a gym. A Smith machine (Technogym S.p.A, Gambettola, Italy) was used to perform the SQ progressive load test [32]. The test was performed following the instructions of Galiano et al. (2020) [32] using a linear encoder (Vitruve, Madrid, Spain) to determine the bar velocity. Three repetitions were performed for light loads (>1.14 m·s^−1^), and two were performed for moderate loads (<1.14 m·s^−1^) [32].

The variable obtained was the estimation of the absolute load moved for the speed of 1 m·s^−1^ through the generation of a quadratic regression line by means of the loads used and the speed at which they were mobilized using Microsoft Excel 2019 (17.0) (Microsoft, Redmon, WA, USA) [33]. We analyzed the R^2^ value to check the prediction accuracy. In all cases, values >0.9 were obtained, which could be considered as values with a high predictive power [34].

The HT protocol [20] was carried out with a player in supine position with his upper back on a bench, placing his feet shoulder width apart and facing slightly outward. The bar was placed on the hips and the player was asked to push the bar concentrically upwards at the maximum possible speed, keeping the pelvis and spine in a neutral position. The rest of the test was performed in the same way as the SQ test.

#### 2.3.3. Acceleration and Speed

The test was performed with appropriate footwear on the artificial turf of a football pitch. Acceleration capacity was assessed through the 10 m sprint test with the start being from a standstill; while the maximum speed was assessed through the 30 m sprint test [35,36,37]. The assessment was carried out by placing 3 single-beam timing gate units (Witty, MICROGATE, Bolzano, Italy), at the start, 10 m, and 30 m, respectively; these were placed at a height of 0.75 m and facing each other at a distance of 1.5 m. Players started the tests 0.5 m from the starting timing gate. Three sprints were performed, recording for analysis the best value in seconds of each attempt, measured with an accuracy of 0.01 s each time [36].

#### 2.3.4. Change-of-Direction Ability

The test was performed with appropriate footwear on the artificial turf of a football pitch. This test assesses the players’ ability to change direction. The assessment was carried out on artificial turf using specific football shoes. The test was performed by following the instructions of Gonzalo-Skok et al. (2015) [22], using 2 single-beam timing gate units (Witty, MICROGATE, Bolzano, Italy). Two repetitions were performed, and the best time (in seconds) was used for subsequent analysis [22].

#### 2.3.5. Cardiorespiratory Fitness

The test was performed with appropriate footwear on the artificial turf of a football pitch. The assessment of cardiorespiratory fitness was assessed with the 30–15 IFT [38], a test suitable for the assessment of team sports athletes [27]. All players on each team performed the protocol at the same time. The protocol was carried out according to the instructions given in the study by Buchheit (2008) [38]. The speed reached in the intermittent test (V_IFT_) was recorded.

### 2.4. Statistical Analyses

Descriptive statistics are presented as the mean ± standard deviation by PHV groups at test and retest. A one-way ANOVA with Bonferroni correction was conducted to assess differences in demographic, anthropometric, and physical performance variables across groups of PHV.

The *t*-test and the intraclass correlation coefficient (ICC) were used to investigate the reliability of the tests evaluated, comparing the test and the retest scores. The ICC is commonly used to describe relative reliability (i.e., the consistency of measurements in individuals in one group relative to others). An ICC < 0.8 was considered insufficient, values between 0.8 and 0.9 moderate, and values > 0.9 were considered high [39].

To improve the accuracy of our statistical analysis, we use other error measures to assess the differences between test (T1) and retest (T2). It is understood that the smaller the error value the smaller the dispersion between measurements.

The sum of squared errors (SSE), mean sum of squared errors (MSE), root mean sum of squared errors (RMSE), and percentage error were calculated, and the standard error (SEE) of estimation was calculated as shown [40,41].

Absolute reliability (the consistency of repeated measurements for individuals) was analyzed through the calculation of the standard error of measurement (SEM) as a percentage of the mean value of the measurements. The SEM is used to quantify the precision of individual scores on a test, without being influenced by inter-individual variability. A value ≤15% is considered acceptable [40]. We also use %SEM = mean of the difference scores between 2 trials × 100/mean of the first trial, and the coefficient of variation (CV) was also calculated.

We used the CV method, which provides useful information in the presence of heteroscedasticity. We consider the reliability to be acceptable when CV ≤ 10% [41].

The minimum detectable change at 90% (MDC90) of the players was calculated to determine the smallest change that must be observed in the test to consider it a real change in performance. This was calculated as below.

Finally, Bland–Altman plots were used to assess the reproducibility of the tests [42], using an ANOVA test for repeated measures to calculate the difference. Similarly, Cohen’s d was calculated to quantify the magnitude of the difference between T1 and T2 [43].

Heteroscedasticity was assessed using ANOVA. Initially, the mean value of both measurements for each test was calculated, and this variable was divided into performance quartiles. These quartiles were included as an independent variable. The absolute value (negative values multiplied by −1) of the difference between the two evaluations of each test (retest–test) was included as the dependent variable.

Statistical analyses were performed using version 25.0 of SPSS statistical software for MacOS (IBM, Armonk, NY, USA), setting the level of significance at *p* < 0.05.

## 3. Results

The final sample size was composed of 197 young trained football players (14.74 ± 2.2 years old). The descriptive characteristics of the players distributed by maturational age are shown in Table 1. Analysis revealed a statistically significant difference (all *p* < 0.05) between maturity groups in most of the tests regardless of the time of evaluation (test–retest), showing the higher performance of PHV2 in relation to PHV1 and PHV0, and PHV1 with respect to PHV0 (Table 1). The only non-significant differences were evident between the PHV1 and PHV2 groups in leg length, 30–15 IFT, and V–cut test (all *p* > 0.05) in both T1 and T2. Similarly, no significant differences between PHV0 and PHV1 were evident for 30–15 IFT (*p* > 0.05).

The test–retest reliability of all the field-based fitness test are shown in Table 2. In the total sample, significant differences were found between T1 and T2 in the CMJ (−0.014 ± 0.6 cm, *p* = 0.001), 10 m sprint test (−0.01 ± 0.04 s, *p* < 0.001), and V–cut test (−0.02 ± 0.07 s, *p* < 0.001) and in the progressive loading test for SQs (−0.66 ± 2.71 kg, *p* = 0.009). The effect sizes (Cohen *d*) of the mean differences were 0.03, 0.09, 0.04, and 0.06, respectively. In the rest of the evaluated tests, non-significance differences were found between T1 and T2 (*p* > 0.05). The ICCs reported a high reproducibility, ranging from 0.97 to 0.99 (all *p* < 0.01) in all tests.

All the field-based fitness tests showed small error rates (%error = 1.82–10.69; %SEM = 1.82–7.73; %CV = 4.39–9.39), except for the values obtained in the CMJ, SQ, and HT (%CV = 15.2–21.11). All the field-based fitness tests showed an MDC90 close to 0, indicating that there was no real change between T1 and T2.

According to biological age, the reliability of the PHV0 group was similar to the total sample, with the exception that there was a non-significant difference between T1 and T2 in the CMJ (*p* > 0.05, CV = 12.7%). Regarding the PHV1 group, there was only a significant difference between T1 and T2 in the 10 m sprint test (−0.01 ± 0.03 s, *p* = 0.033; ES = 0.125; ICC = 0.97). In the case of the PHV2 group, the reliability analysis followed the same trend as the total sample.

Finally, Bland–Altman plots supported the reliability of all field-based fitness tests, with differences of nearly 0 and narrow limits of agreement (LoA). Except for CMJ (*p* = 0.037), heteroscedasticity was not observed in any of the assessed tests, based on the absence of differences between performance quartiles (all *p* > 0.05) within each test. The linear regression model showed proportional bias in CMJ (r = 0.161, R^2^ = 0.026, *p* = 0.024) and the 30 m sprint (r = 0.227, R^2^ = 0.052, *p* = 0.001) tests, indicating that the better the performance, the larger the difference between trials.

## 4. Discussion

The aim of this study was to analyze the reliability of a field-based fitness test battery in young trained football players (11–18 years) according to biological age. The main finding was that all the field-based fitness tests were reliable for the general sample as well as for the biological age groups, meaning that this variable does not influence the reliability of the field-based fitness tests assessed.

All the field-based fitness tests seemed to be reliable, although the CMJ, 10 m sprint, V–cut test, and the progressive loading test for SQ showed significant differences between T1 and T2. However, these tests presented a trivial effect size (<0.2) [44] and a high ICC ranging from 0.97 to 0.99, which indicates the high reproducibility of these tests. In addition, all the field-based fitness tests presented low measurement errors [39], except for the CMJ test and the progressive loading test for SQ and HT, which reported a %CV > 10.

No significant differences were found for any of the age groups between T1 and T2 in the 30 m sprint test, and a reproducibility between groups of different biological age with no significance between T1 and T2 and with very good ICC (PHV0 = 0.94; PHV1 = 0.97; PHV2 = 0.94) it was observed, as was the case with other authors who, in their studies, arrived at an ICC between 0.77 and 0.98 [25] for adult football players. Moreover, no such significant inter-day differences were obtained in the 30–15 IFT, again finding a high test reliability independent of biological age as shown by the ICCs (PHV0 = 0.98; PHV1 = 0.96; PHV2 = 0.98). These results are in line with those obtained in other studies, with ICCs ranging from 0.80 to 0.99 for adult athletes [26]. Similarly, no significant differences were obtained in the progressive loading test for HT, independent of biological age, presenting CCIs that confirmed the high reproducibility of the test (PHV1 = 0.98; PHV2 = 0.99). Inter-day reliability has never been assessed for this test before, so comparisons are not possible. However, other tests, such as the power clean in a rugby league player population [45], have been shown to be reliable for assessing lower-body strength showing no significant differences in their inter-day assessment. Therefore, it seems that 30 m sprint test, the 30–15 IFT, and the progressive loading test for HT are reliable in a similar way to what has been observed in the literature, with biological age having no influence on the reliability of the tests.

The rest of the tests (i.e., the CMJ, 10 m sprint, V–cut test, and the progressive loading test for SQ) showed significant differences between T1 and T2 in all three biological age groups, with the sole exception of the V–cut test in the PHV1 group (*p* = 0.462). Significant differences in the inter-day assessment (*p* < 0.001 and *p* < 0.01, respectively) were showed for the CMJ, but with a 0.99 ICC in the three groups. These results are in line with those obtained in other studies [23,24] of young trained football players. As in our case, those studies presented an ICC between 0.96 and 0.99, a CV between 4.79% and 6.76% [23], and an ICC of 0.87 and a CV of 2.2% [24], showing a high reproducibility. Similarly, the reliability of the progressive loading test for SQ was tested in a single study in resistance-trained men (age 25.0 ± 3.9 years) [21], showing, significant differences between T1 and T2, but with an ICC ranging from 0.61 to 0.97 and a CV of 2.9%, a sign of good reproducibility, following a similar trend to our results, where we found very good ICCs regardless of biological age (PHV1 = 0.97; PHV2 = 0.98). On the other hand, the V–cut test in basketball players aged 12–20 years [22] and the 10 m sprint test in adult football players aged 17–25 years [25], reported no significant differences between T1 and T2 (both *p* > 0.01). However, in another study with young trained football players [24], significant differences were found between T1 and T2 (*p* < 0.1), showing an ICC of 0.87 and a CV of 2.2%, thus demonstrating the high reproducibility of the 10 m sprint test. The fact that no significant differences were found between these studies (except in the last one) and our, it could be due to the fact that these samples had greater sporting experience as well as a greater chronological age, with our sample being younger and more inexperienced. Also, in our study, significant differences may be due to type I errors, because the sample is larger than previous studies and the mean changes between T1 and T2 are quite small. Moreover, it should be noted that, those tests in which there were significant differences between T1 and T2, also presented statistical variables that reinforce the good reproducibility of the tests themselves (i.e., ICC and error measures). The differences in the measures recorded between T1 and T2 did not exceed 0 cm for PHV0, 0.01 cm for PHV1, and 0.2 cm for PHV2 in the CMJ test; 0.01 s for PHV0, 0.02 s for PHV1, and 0.01 s for PHV2 in the 10 m sprint test; more than 0.02 s for PHV0 and 0.02 s for PHV2 in the V–cut tests; and 0.58 kg for PHV1 and 0.69 kg for PHV2 in the progressive loading test for SQ. This is minimal in terms of performance and the interpretation of the data. In addition, except for CMJ, heteroscedasticity analyses indicated that the tests’ reliability was independent of the performance level in the three groups, thereby justifying its applicability across athletes of varying expertise levels. For all these reasons, we could confirm that there was good test–retest reliability and we were able to rule out the learning effect on the retest measurements.

However, the CMJ test and progressive loading tests for SQ and HT showed a CV greater than 10% for the three groups (CMJ: PHV0 = 12.7; PHV1 = 16.6; PHV2 = 12.3; SQ: PHV1 = 15.9; PHV2 = 15.2, HT: PHV1 = 20.9; PHV2 = 20.5), which could indicate that the reliability of these tests was questionable [41]. This could be due to the fact that the three tests are the most complex in terms of technique. The literature has shown how, in jumps such as the CMJ, kinematic variables related to technique, such as the degree of joint flexion (i.e., ankle, knee, and hip flexion), may influence the force production capacity and performance [46]. Similarly, other authors have shown how technical aspects also influence performance by varying even the muscle activation in SQ and HT tests [46,47]. Slight variations in the technique of these three tests could explain those values obtained in the CV. Nevertheless, the test’s responsiveness could help us to understand the common changes developed in the above-mentioned tests (greater than the CV) to know whether they can be used to monitor player’s progression throughout the season and their development [22]. We also could add that, during adolescence, experiencing repeated growth changes alters the length of the segments in an unexpected way, which causes in subjects a process of motor re-calibration to adapt their “new body” to the demands of the movement patterns [48], a situation that could alter the technical capacities of people in periods of biological development and close to this moment, as is the case with our sample.

In reference to the PHV, despite its proven relationship with performance in different aspects of a footballer’s physical fitness [15], its action as a conditioning variable in the reliability of the tests had no effect. Therefore, the tests evaluated are reliable regardless of the biological age of the football players. One study [23] observed an upward gradient in the reliability of the CMJ assessment as biological age increased, something that may have developed with the maturity and sporting experience of the players. However, despite this trend, reliability was acceptable regardless of whether pre-, circa-, or post-PHV. Similarly, other authors [17] observed that, in braking variables during the CMJ assessment, there was very little variation between ages from 11 to 16 years. The results obtained by Buchheit and Mendez-Villanueva (2013) in anthropometric variables, the CMJ, and 10 m sprint tests reinforce this idea, reporting a high reliability in the tests regardless of chronological and biological age. Although there are hypotheses that could lead us to think that the assessments with pre-PHV players could present poor reliability due to the phenomenon of “adolescent awkwardness” [16], regular and correctly structured training (i.e., such as that undertaken by the pre-PHV players in our sample as a consequence of the correct training content) [12] could stabilize locomotor functions and mitigate possible differences in reliability between the maturational age groups.

The results of the present study not only provide valuable information on the reliability of the proposed field-based fitness test battery, but also provide reference values that could be useful for talent identification. There is a deficit in the literature regarding reference values for the level of physical fitness of young soccer players; however, it has been shown that parameters such as lower-body explosive strength [5,6,49], COD ability [6], or cardiorespiratory fitness [7,50] seem to be determinants of performance and indicators for early detection of talent [51], so this study provides reference values for this purpose.

The main strength of this work was the fact that it presented a large sample, which is often difficult to achieve with a sports population. The evaluation of a large number of tests that give a complete picture of the physical fitness of the players was also a strength. Furthermore, the field-based test battery proved its reliability in taking into account the biological age of the players, something that has not previously been achieved in the literature to this extent. In this sense, another strength of this study that gives more value to the results was that it demonstrated the reliability of a test as accessible and simple to perform as the CMJ, which is a great indicator of fatigue during the microcycle and has a direct relationship with other performance variables such as repeat sprint capacity [52]. Finally, although the ICC, Bland–Altman, SEM, and CV are the most commonly used statistics to report reliability in sport performance, we added other statistical variables such as errors to provide a more complete analysis of reliability. The main limitation of our study is that the familiarity with strength training and the tests developed by our sample was low; perhaps it would be interesting to assess a population with a higher degree of experience. Another limitation was not extrapolating the results to include a population of female soccer players.

## 5. Conclusions

This study was designed to analyze the reliability of the current field-based tests battery for assessing physical fitness in young trained football players (11–18 years), according to biological age. The results of this study indicate that all the field-based fitness tests (i.e., anthropometric measurements, the CMJ test, the progressive loading test for SQ and HT, the 10 m and 30 m sprint tests, the V–cut test, and the 30–15 IFT) are reliable in terms of estimating physical fitness in young trained football players (11–18 years), without being influenced by biological age. Soccer professionals could benefit from this field-based fitness test battery to longitudinally assess the performance development of their players and make decisions regarding their training process over time. However, more research is needed to investigate this topic with other tests and in other populations like female and senior male soccer players.

## Figures and Tables

**Table 1:** Descriptive characteristics of the sample by PHV groups at test and retest.

	All	PHV0	PHV1	PHV2	PHV0 vs. PHV1	PHV0 vs. PHV2	PHV1 vs. PHV2
Test							
Age (y)	15 (2.2)	12 (1.12)	15 (0.76)	16 (1.2)	<0.001	<0.001	<0.001
	(12–19)	(12–13)	(13–15)	(16–19)			
Weight (kg)	59 (13.8)	43.13 (7.57)	60.28 (4.24)	69.51 (7.24)	<0.001	<0.001	<0.001
	(34.5–84.4)	(34.5–44.6)	(46.7–69.6)	(57.3–84.4)			
Height (cm)	166.88 (12.85)	152.64 (9.05)	168.62 (5.08)	176.13 (6.23)	<0.001	<0.001	<0.001
	(139.1–195)	(139.1–159.2)	(153.5–176.3)	(164.3–195)			
Sitting height (cm)	84.38 (6.77)	76.87 (4.27)	85.7 (2.69)	89.05 (3.95)	<0.001	<0.001	<0.001
	(69.5–100.7)	(69.5–82.1)	(74.3–90.1)	(76.1–100.7)			
Leg length (cm)	82.51 (11.49)	76.9 (12.85)	82.92 (3.76)	87.07 (5.98)	0.01	<0.001	0.174
	(66.8–94)	(66.8–82.5)	(68.2–90.2)	(74.4–94)			
CMJ (cm)	32.33 (5.61)	27.66 (3.51)	31.78 (5.29)	35.73 (4.38)	<0.001	<0.001	<0.001
	(22.3–60.6)	(22.3–31.5)	(29.6–37.2)	(31.9–60.6)			
10 m sprint (s)	1.97 (0.15)	2.14 (0.12)	1.95 (0.09)	1.87 (0.08)	<0.001	<0.001	0.012
	(1.64–2.44)	(2.1–2.36)	(1.71–2.3)	(1.64–2.02)			
30 m sprint (s)	4.74 (0.43)	5.22 (0.34)	4.62 (0.25)	4.46 (0.17)	<0.001	<0.001	<0.001
	(3.91–6.09)	(5.13–6.09)	(4.13–4.65)	(3.91–4.56)			
VCUT (s)	7.25 (0.55)	7.73 (0.59)	7.02 (0.37)	7 (0.32)	<0.001	<0.001	1.00
	(6.43–8.56)	(7.39–8.56)	(6.51–7.2)	(6.43–7.18)			
30–15 (km/h)	19.5 (1.08)	19 (0.87)	19.37 (1.04)	19.75 (1.09)	0.48	0.001	0.257
	(16.5–23)	(16.5–20)	(18.5–23)	(18.5–23)			
SQ (kg)	75.11 (11.96)	-	69.61 (11.4)	76.5 (11.68)	<0.001	<0.001	0.02
	(30–90)	(–)	(45–85)	(65–90)			
HT (kg)	74.43 (15.8)	-	65.97 (14.43)	76.33 (15.64)	<0.001	<0.001	0.009
	(25–90)	(–)	(40–85)	(55–90)			
Retest							
Age (y)	15 (2.23)	12 (1.13)	15 (0.76)	17 (1.24)	<0.001	<0.001	<0.001
	(12–19)	(12–13)	(13–15)	(16–19)			
Weight (kg)	59.01 (13.85)	43.17 (7.6)	60.16 (4.33)	69.54 (7.22)	<0.001	<0.001	<0.001
	(34.2–85)	(34.2–44.7)	(46.3–69)	(57.5–84.4)			
Height (cm)	166.92 (12.9)	152.64 (9.05)	168.6 (5.06)	176.22 (6.31)	<0.001	<0.001	<0.001
	(139.4–195)	(139.4–159.4)	(153.2–176.5)	(164.3–195)			
Sitting height (cm)	84.37 (6.77)	76.9 (4.28)	85.68 (2.69)	89.04 (3.95)	<0.001	<0.001	<0.001
	(69.5–100.7)	(69.5–82.56)	(74.2–90.1)	(76.1–100.7)			
Leg length (cm)	82.56 (11.5)	76.9 (12.84)	82.92 (3.75)	87.17 (6.09)	0.006	<0.001	0.066
	(66.6–94)	(66.6–82.1)	(68.2–90.3)	(74.5–94)			
CMJ (cm)	32.46 (5.71)	27.66 (3.54)	31.79 (5.36)	35.97 (4.42)	<0.001	<0.001	<0.001
	(22.5–60.5)	(22.5–31.4)	(29.8–37)	(31.6–60.5)			
10 m sprint (s)	1.96 (0.16)	2.12 (0.13)	1.94 (0.92)	1.86 (0.09)	0.002	<0.001	<0.001
	(1.64–2.4)	(2.1–2.4)	(1.7–2.3)	(1.64–2)			
30 m sprint (s)	4.47 (0.43)	5.23 (0.39)	4.61 (0.24)	4.44 (0.21)	0.003	<0.001	0.023
	(3.9–6.1)	(5.14–6.1)	(4.12–4.68)	(3.92–4.51)			
VCUT (s)	7.23 (0.55)	7.71 (0.58)	7.01 (0.37)	6.98 (0.33)	<0.004	<0.001	1.00
	(6.41–8.51)	(7.42–8.51)	(6.57–7.25)	(6.41–7.05)			
30–15 (km/h)	19.55 (1.07)	18.97 (0.84)	19.46 (1.01)	19.78 (1.09)	0.186	<0.001	0.437
	(16.5–23)	(16.5–20)	(18.5–23)	(18.5–23)			
SQ (kg)	74.45 (11.75)	-	69.02 (10.91)	75.81 (11.54)	-	-	0.02
	(30–90)	(–)	(45–85)	(65–90)			
HT (kg)	74.32 (15.48)	-	66.79 (13.78)	76.06 (15.39)	-	-	0.016
	(25–90)	(–)	(40–85)	(55–90)			

Values are presented as means (standard deviation). CMJ, counter movement jump; HT, hip thrust; 30–15, 30–15 intermittent fitness test; SQ, squat; VCUT, V–cut test. Players were classified according to their PHV as pre < −1 (PHV0 group), mid from −1 to 1 (PHV1 group), and post > 1 (PHV2 group) [29].

**Table 2:** The test–retest reliability of the field-based fitness tests by PHV group at retest.

	n	Intertrial Difference (Retest-Test)	*p*-Value	Cohen’s d	ICC (95% CI)	SSE	MSE	RMSE	% Error	%SEM	MDC90	% CV	SEE
All													
CMJ (cm)	197	0.14 ± 0.60	0.001	0.025	0.997	74.203	0.377	0.614	1.821	0.433	0.010	17.469	0.597
10 m sprint (s)	197	−0.01 ± 0.04	<0.001	0.088	0.979	0.409	0.002	0.046	6.418	0.467	0.011	8.085	0.044
30 m sprint (s)	197	−0.01 ± 0.12	0.521	-	0.981	2.965	0.015	0.123	6.196	0.119	0.003	9.396	0.123
VCUT (s)	197	−0.02 ± 0.07	<0.001	0.036	0.996	1.035	0.005	0.072	2.681	0.274	0.006	7.676	0.070
30–15 (km/h)	162	0.03 ± 0.26	0.181	-	0.985	11.250	0.069	0.264	4.054	0.142	0.003	5.519	0.262
SQ (kg)	118	−0.66 ± 2.71	0.009	0.055	0.986	915.356	7.757	2.785	4.262	0.864	0.020	15.693	2.680
HT (kg)	118	−0.10 ± 2.73	0.687	-	0.989	874.839	7.414	2.723	2.443	0.113	0.003	21.113	2.671
PHV 0													
CMJ (cm)	68	0.05 ± 0.32	0.248	-	0.998	7.113	0.105	0.323	1.965	0.165	0.004	12.716	0.324
10 m sprint (s)	68	−0.01 ± 0.06	0.037	0.116	0.944	0.218	0.003	0.057	10.690	0.666	0.016	5.741	0.056
30 m sprint (s)	68	0.01 ± 0.14	0.742	-	0.961	1.376	0.020	0.142	9.482	0.110	0.003	6.977	0.143
VCUT (s)	68	−0.03 ± 0.07	0.001	0.048	0.996	0.352	0.005	0.072	3.126	0.361	0.008	7.530	0.067
30–15 (km/h)	35	−0.03 ± 0.21	0.422	-	0.985	1.500	0.043	0.207	5.175	0.150	0.004	4.505	0.205
PHV 1													
CMJ (cm)	30	0.01 ± 0.90	0.937	-	0.993	23.354	0.778	0.882	3.834	0.041	0.001	16.620	0.913
10 m sprint (s)	30	−0.01 ± 0.03	0.033	0.125	0.973	0.028	0.001	0.030	7.996	0.595	0.014	4.752	0.029
30 m sprint (s)	30	0.00 ± 0.04	0.440	-	0.993	0.047	0.002	0.039	3.860	0.123	0.003	5.239	0.039
VCUT (s)	30	−0.01 ± 0.08	0.462	-	0.990	0.168	0.006	0.075	4.436	0.146	0.003	5.264	0.077
30–15 (km/h)	28	0.09 ± 0.39	0.232	-	0.963	4.250	0.152	0.390	7.792	0.461	0.011	5.260	0.381
SQ (kg)	19	−0.59 ± 3.41	0.462	-	0.977	216.536	11.397	3.376	6.816	0.846	0.020	15.928	3.351
HT (kg)	19	0.83 ± 2.91	0.232	-	0.989	165.019	9.168	3.028	5.533	1.251	0.029	20.979	2.834
PHV 2													
CMJ (cm)	99	0.24 ± 0.62	<0.001	0.055	0.994	43.736	0.442	0.665	2.329	0.681	0.016	12.271	0.625
10 m sprint (s)	99	−0.01 ± 0.04	<0.001	0.170	0.942	0.163	0.002	0.041	9.020	0.783	0.018	4.627	0.038
30 m sprint (s)	99	−0.01 ± 0.12	0.287	-	0.887	1.543	0.016	0.125	13.871	0.301	0.007	4.393	0.125
VCUT (s)	99	−0.02 ± 0.07	0.016	0.053	0.988	0.515	0.005	0.072	3.913	0.247	0.006	4.664	0.071
30–15 (km/h)	99	0.03 ± 0.23	0.202	-	0.988	5.500	0.056	0.236	4.285	0.153	0.004	5.521	0.235
SQ (kg)	99	−0.69 ± 2.59	0.010	0.057	0.987	698.821	7.059	2.657	4.066	0.867	0.020	15.274	2.561
HT (kg)	99	−0.27 ± 2.69	0.315	-	0.992	709.820	7.170	2.678	2.403	0.340	0.008	20.532	2.647

ICC, intraclass correlation coefficient; CI, confident interval; SSE, sum of squared errors; MSE, mean sum of squared errors; RMSE, root mean sum of squared errors; % error, percentage error; %SEM, standard error of measurement; % CV, percentage coefficient of variation; SEE, standard error of estimate. CMJ, counter movement jump; HT, hip thrust; 30–15, 30–15 intermittent fitness test; SQ, squat; VCUT, V–cut test.

## Data Availability

The data supporting these findings can be obtained from the lead author, J.J.-I., upon request.

## Supplementary Materials

The following supporting information can be downloaded at: https://www.mdpi.com/article/10.3390/life14111448/s1, Table S1: Sample characteristics.
