# Supplementary material for: Age-Related Differences and Reliability of a Field-Based Fitness Test Battery in Young Trained Footballers: The Role of Biological Age
Source: Life (Basel). 2024 Nov 8;14(11):1448. doi: 10.3390/life14111448 (PMC11595939; doi:10.3390/life14111448)
Supplement: Supplementary file 1 [file life-14-01448-s001.zip › life-3261649-supplementary.pdf]

Supplementary Material

Supplementary Table S1. Sample characteristics.

Category	Sample size	CA (years)	BA (PHV)	FE (years)	STE (years)
U-13	32	11.25±0.54	-2.46±0.59	5-6	0
U-15	48	13.34±0.59	-0.91±1.94	7-8	0
U-17	65	15.32±0.53	1.33±0.53	8-9	1-2
U-19	54	17.35±0.8	2.1±0.77	9-10	1-2

BA: Biological age; *CA:* Chronological age; *FE:* Football Experience; *STE:* Strength Training Experience.
